# Effect of Stable and Metastable Phase Microstructures on Mechanical Properties of Ti-33Nb Alloys

**DOI:** 10.3390/ma18102351

**Published:** 2025-05-18

**Authors:** Shitao Fan, Yingqi Zhu, Na Min

**Affiliations:** 1 School of Materials Science and Engineering, Shanghai University, Shanghai 200444, China; stfan@shu.edu.cn (S.F.); zyq_@shu.edu.cn (Y.Z.); 2 Laboratory for Microstructures, Shanghai University, Shanghai 200444, China

**Keywords:** Ti-33Nb alloy, superelasticity, microstructure, mechanical properties

## Abstract

In this paper, the crystal structure, microstructure, and deformation behavior in the Ti-33Nb alloy under furnace-cooling (FC) and water-quenching (WQ) conditions after holding at 950 °C for 0.5 h are reviewed. The stable and metastable phases obtained under FC and WQ heat treatments have significantly different influences on the mechanical properties of this alloy. The furnace-cooling specimens possess a β and α phase at room temperature, while water-quenched specimens are composed of a metastable β phase and martensite α″ phase. According to the results of the nanoindentation test, the hardness value of the FC specimens is 2.66 GPa, which is lower than that of the WQ specimens. It can be attributed to the presence of a large number of α phases. The indentation depth recovery ratio (η_h_) and work recovery ratio (η_w_) of the WQ specimens are 19.02% and 19.54%, respectively, indicating a better superelastic response than the FC specimens. In addition, the wear resistance (H/E_r_) and yield pressure ($H^{3}/E_{r}^{2}$) of the WQ specimens are 0.0282 and 0.0030 GPa, respectively, suggesting a better wear resistance and resistance of plastic deformation.

## 1. Introduction

Titanium-based alloys have garnered significant attention in the field of biomedical materials, owing to their exceptional biocompatibility, superior corrosion resistance, favorable mechanical properties, and low Young’s modulus [1,2,3]. Among these, the commercially available Ti-6Al-4V alloy has been extensively utilized in applications such as hard tissue implantation and soft tissue interventional therapy [4,5]. However, the release of Al and V ions from this alloy into the human body poses potential health risks [6]. Specifically, V ions exhibit cytotoxicity, which can induce adverse reactions in human tissues, while Al ions have been associated with neurological disorders, including Alzheimer’s disease [7]. Furthermore, despite the relative low elastic modulus of titanium alloys (120 GPa) compared to traditional medical metals such as stainless steel (210 GPa) and Co-Cr alloys (240 GPa), it remains substantially higher than that of human bone (10–30 GPa). This mismatch in elastic modulus can lead to the stress-shielding phenomenon, resulting in bone resorption and eventual implant failure [8,9,10]. To address these long-term safety concerns, researchers are actively developing novel titanium alloys that exclude Al and V elements, with a particular focus on enhancing strength and further reducing the elastic modulus of these materials.

In recent decades, metastable β-Ti alloys based on the binary Ti-Nb system have attracted significant attention due to their low Young’s modulus, high specific strength, and intrinsic corrosion resistance [11,12]. These alloys offer numerous advantages, primarily stemming from their ability to achieve a wide range of mechanical properties through the precise control of phase transformations during thermomechanical processing. Titanium undergoes allotropic transformations during the body-centered cubic (bcc) β phase to the hexagonal close-packed (hcp) α phase at 1156 K [13]. Niobium (Nb) is commonly added as a β-stabilizing element to Ti alloys, enabling a detailed investigation of the bcc β → α′/α″ martensitic transformation. Within the Nb-Ti binary system, only three stable phases exist, which are the liquid, bcc β phase, and hcp α phase [14]. However, the β → α transformation is kinetically sluggish, facilitating the occurrence of various metastable transformations. At room temperature, Ti-Nb alloys can exhibit two non-equilibrium phases, namely hexagonal α′ martensite and orthorhombic α″ martensite, both of which form during rapid quenching from the β phase. Additionally, the hexagonal ω phase may precipitate during slow quenching or isothermal aging [15]. These metastable transformations, along with the factors influencing their competitive formation within a metastable β matrix, have been extensively studied [16]. Notably, the α′/α″ composition boundary in Ti-Nb binary alloys has been identified at Ti-13.85Nb wt.% [17]. The superelasticity and shape memory behavior observed in metastable β-Ti alloys are attributed to the reversible stress-induced martensitic transformation between the parent β phase (bcc) and the orthorhombic α″ martensite phase [18]. Kim et al. [19] reported that the shape memory effect is present in Ti-(22–25) at. % Nb alloys, while superelastic recovery occurs in Ti-(25.5–27) at. % Nb alloys at room temperature. However, the superelastic strain of metastable β-type Ti-Nb alloys is generally lower than that of commercially available biomedical Ni-Ti alloys, primarily due to the low critical stress for slip deformation [20]. This limitation poses a significant challenge for the application of Ti-Nb alloys in the biomedical field. During cooling from the high-temperature β phase to room temperature (RT), titanium alloys can undergo either a displacive β → α″ martensitic transformation or a diffusive β → β + α transformation, depending on the cooling rate [21]. At high cooling rates, the microstructure predominantly consists of a combination of β and α″ martensite phases. Ti-Nb alloys with a significant volume fraction of the α″ phase exhibit enhanced strength while maintaining high ductility and low elastic modulus [22]. Conversely, at low cooling rates, wider α (hcp) bands form within the α/β colonies [23]. The lamellar β + α structure is known to exhibit high fatigue crack resistance and fracture toughness [21]. Therefore, it can be concluded that cooling rates play a critical role in controlling phase transformations and consequently tailoring the mechanical properties of Ti alloys.

The primary objective of this study is to systematically investigate the mechanical properties of a Ti-33Nb alloy under various heat treatment conditions, with a focus on optimizing its performance for biomedical applications.

## 2. Materials and Methods

**Figure 1:**
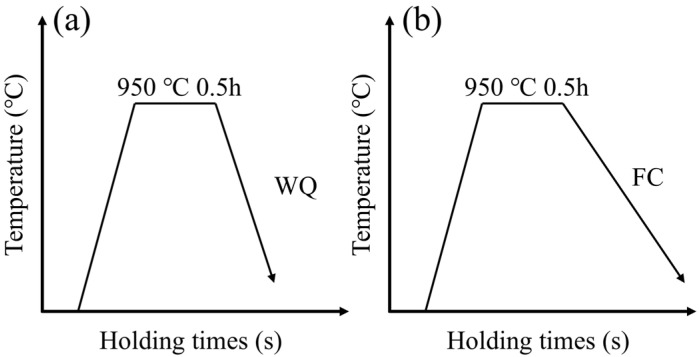
The schematic of the heat treatment process: (**a**) WQ; (**b**) FC.

The Ti-33Nb (wt.%) ingots were fabricated using high-purity titanium and niobium as raw materials in a non-consumable vacuum arc melting furnace in an argon atmosphere. To ensure macroscopic homogeneity of the alloy composition, each ingot was inverted and remelted at least 5 times. Following melting, the ingots were homogenized at 950 °C for 20 h and subsequently cross-sectioned to 10 mm lengths. These sections were then cold-rolled to achieve a 70% reduction in thickness, resulting in strips with a final thickness of 3 mm. Prior to solution treatment, the cold-rolled sheets were cut into specimens using an electro discharge machine. The specimens were ultrasonically cleaned with ethanol and encapsulated in quartz tubes under an argon atmosphere to prevent oxidation during heat treatment. The encapsulated specimens were then subjected to heat treatment at 950 °C for 1.8 ks (Nabertherm 30–3000, Anklam, Germany). After heat treatment, the specimens were quenched into water (WQ) by breaking the quartz tubes or furnace-cooled (FC) for comparison. Figure 1 shows the schematic of the heat treatment process.

The phase constitutions of the samples were characterized using an X-ray diffractometer (XRD, smartlab, Rigaku, Tokyo, Japan) with Cu Kα radiation at a scanning rate of 5°/min under room temperature conditions. A microstructural analysis of both water-quenched (WQ) and furnace-cooled (FC) specimens was performed using scanning electron microscopy (SEM, ZEISS SUPRA 40, ZEISS, Oberkochen, Germany) and transmission electron microscopy (TEM, JEOL JEM-F200, JEOL, Tokyo, Japan). Electron backscatter diffraction (EBSD) measurements were carried out on a SEM (TESCAN Mira 3, TESCAN, Brno, Czech Republic) equipped with HKL Channel 5 software. For SEM observation, the specimens were prepared using standard metallographic procedures (GB/T 13298-2015 [24]), which included grinding up to 2000 grit with SiC paper followed by polishing with a colloidal silica suspension and subsequent etching with a solution 5 vol% HF, 5 vol% HNO_3_, and 90 vol% H_2_O. EBSD samples were prepared by electropolishing in an acid solution of 10 vol% HClO_4_ and 90 vol% methanol for 30 s. Thin foils for TEM analysis were prepared via twin-jet electropolishing using a solution of 10 vol% sulfuric acid and 90 vol% methanol at a temperature of −30 °C and an applied potential of 20 V.

The shape memory and superelastic properties of the alloys were evaluated through a nanoindentation test. The tests were conducted at temperature using a Hystron-Tl950 Tribo-Indentor system (Bruker, MA, USA) equipped with a Berkovich indenter. Indentation marks were created under an applied load of 10 mN with a dwell time of 5 s. The reduced elastic modulus (E_r_) and hardness (H) were automatically calculated using the TriboScan™ software (https://www.pubcompare.ai/product/jzDiCZIBPBHhf-iFoGcl/, accessed on 17 April 2024). Additionally, the superelastic responses, including the depth recovery ratio (η_h_) and work recovery ratio (η_w_), were simultaneously determined during the tests [25]. To ensure the reliability of the results, each sample underwent 10 nanoindentation tests, and final values reported are average of these measurements.

## 3. Results and Discussion

### 3.1. Phase Constituent of Ti-33Nb Alloys

**Figure 2:**
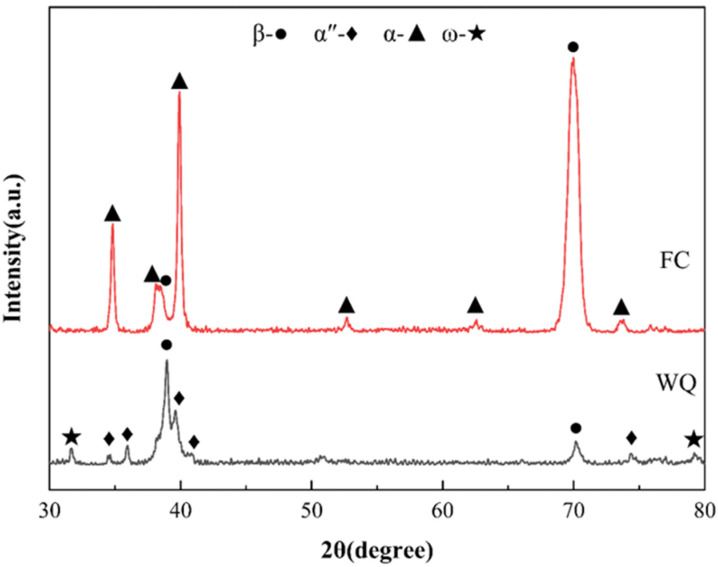
XRD patterns of Ti-33Nb alloys.

The XRD patterns of the water-quenched (WQ) and furnace-cooled (FC) Ti-33Nb alloy specimens are presented in Figure 2. The WQ specimens exhibit reflections corresponding to the β phase and α″ phase. Since the Ti-33Nb alloy lies within the α″ phase region, the presence of the α″ martensitic phase is expected, as confirmed by the XRD results in Figure 2. Additionally, the XRD pattern of the WQ specimens reveals weak diffraction peaks associated with the ω phase, suggesting that a β → β + α″ + ω phase transformation has occurred. It is well-established that the formation of the ω phase typically occurs at relatively slow cooling rates and competes with the formation of the α″ phase [26]. Talbot et al. [27] proposed that both phases can form and grow simultaneously. They suggested that active phonons at high temperatures induce atomic fluctuations, which can become frozen during quenching. Upon rapid cooling, a metastable potential well forms at the ω position, causing atoms to relax towards the ω structure. This mechanism explains the formation of the ω phase. Therefore, the ω phase observed in the WQ specimens is considered metastable. In contrast, the XRD pattern of the FC specimens indicates that the alloy primarily consists of β and α phases. The FC specimens were produced through a slow cooling process, which is generally regarded as a quasi-equilibrium phase transition, leading to a β → β + α transformation [28]. As shown in Figure 2, the XRD patterns demonstrate that a displacive transformation occurred in the WQ specimens, while a diffusional transformation occurred in the FC specimens of the Ti-33Nb alloy.

### 3.2. Microstructure of Ti-33Nb Alloys

**Figure 3:**
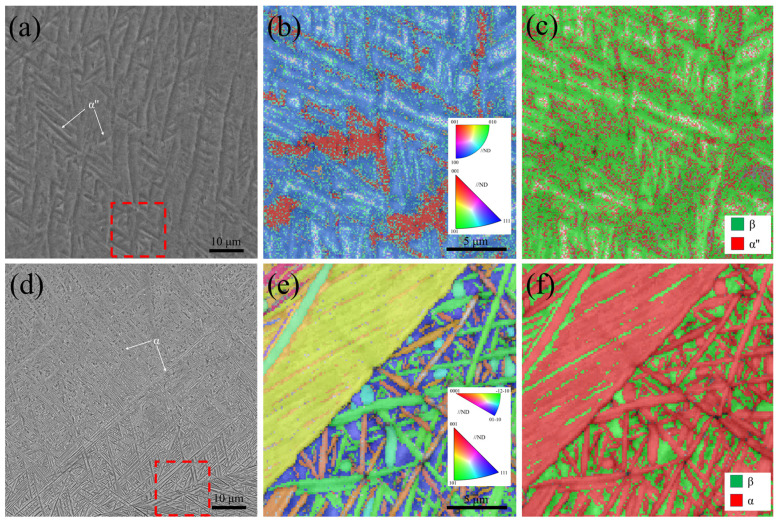
SEM micrographs, EBSD inverse pole figure (IPF) images, and phase maps of the Ti-33Nb alloys: (**a**–**c**) WQ; (**d**–**f**) FC.

As shown in Figure 3, a detailed microstructural analysis of Ti-33Nb alloys under different heat treatment conditions was conducted using scanning electron microscopy (SEM) and electron backscatter diffraction (EBSD). Figure 3a presents the microscopic morphology of the water-quenched (WQ) specimens, revealing a significant number of acicular plates arranged in V-shaped or triangular configurations. This morphology is characteristic of a self-accommodation structure of α″ martensite [29], indicating that rapid cooling from the high-temperature β phase region induced a β → α″ phase transformation. Figure 3b,c displays the EBSD maps of the WQ specimens. The inverse pole figure (IPF) map in Figure 3b demonstrates that the orientations of martensite are relatively uniform, suggesting pronounced variant selection during water quenching. Furthermore, the phase map in Figure 3c confirms the coexistence of β and α″ phases, providing additional evidence of the martensitic transformation.

Figure 3d–f depicts the microstructure of the furnace-cooled (FC) specimens. Figure 3d shows that numerous long needle-like structures are predominantly distributed along the grain boundaries, with fewer located within the grains. This observation indicates that the α phase preferentially nucleates at grain boundaries and subsequently grows into the grain interiors. In the central region of Figure 3e, two large areas with identical orientation colors are observed, corresponding to α/β colonies. The distinct α/β/α structures are clearly visible in the phase map presented in Figure 3f. Previous studies have reported that the slow cooling of duplex titanium alloys from the β phase region can lead to the formation of α/β colony structures [30]. In the case of the FC specimens, the furnace cooling process is sufficiently slow to facilitate a pseudo-equilibrium phase transformation from the β phase to the equilibrium α phase, resulting in the development of these colony structures.

**Figure 4:**
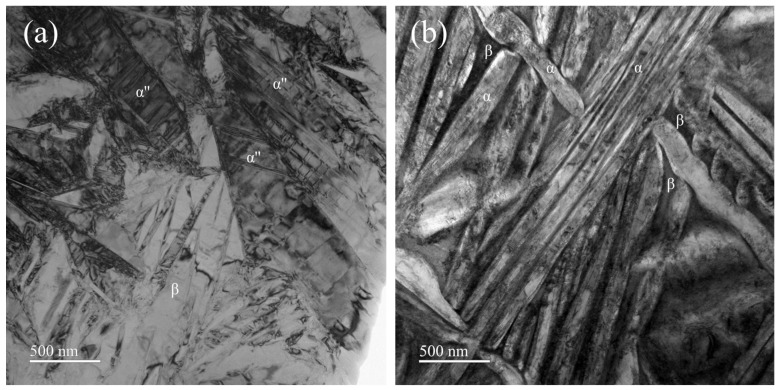
The bright-field (BF) TEM images of the (**a**) WQ and (**b**) FC specimens.

To further elucidate the microstructural details, transmission electron microscopy (TEM) analysis was performed on the water-quenched (WQ) and furnace-cooled (FC) specimens of the Ti-33Nb alloy. Figure 4 presents the bright-field (BF) images of the WQ and FC specimens. Based on the XRD results and EBSD phase maps, the phase composition of the WQ specimen was identified as β + α″, while that of the FC specimen was determined to be β + α. Accordingly, the plate-like structures observed in Figure 4a represent distinct variants of α″ martensite, with their diffraction contrasts arising from variations in orientation. Additionally, lath-like substructures were observed within some of the martensite phases. Figure 4b illustrates a composite microstructure in the FC specimens, consisting of fine bands of β and α phases. The α plates exhibit an average width ranging from tens to hundreds of nanometers, while their length extends to the micrometer scale, resulting in a high aspect ratio. This observation suggests that during the slow cooling process from the β phase region to room temperature, the FC specimens undergo a β → β + α phase transformation. Due to the relatively low cooling rate, the FC specimens experience a more complete phase transition, leading to the formation of coarser α bands.

**Figure 5:**
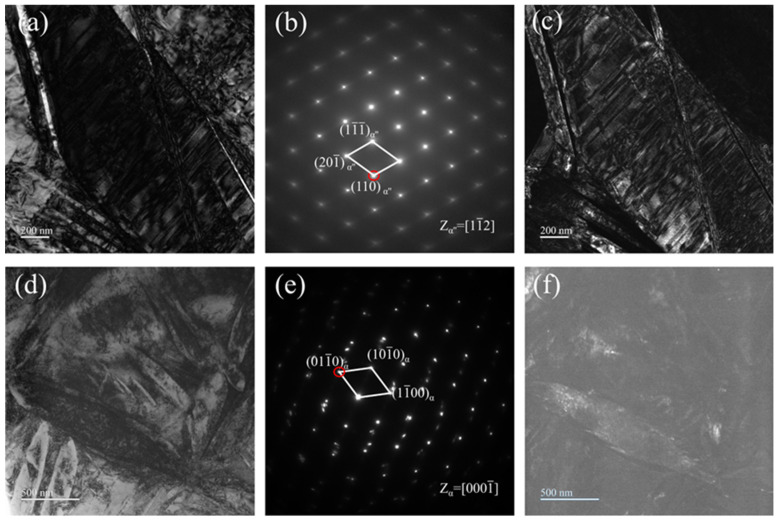
TEM images of the (**a**–**c**) WQ and (**d**–**f**) FC specimens: (**a**,**d**) BF images, (**b**,**e**) selected area electron diffraction (SAED) patterns, and (**c**,**f**) dark-field (DF) image using the spots indicated by red circles in the SAED patterns.

As illustrated in Figure 5, the morphological characteristics of Ti-33Nb alloys were examined using bright-field (BF) images, selected area electron diffraction (SAED), and dark-field (DF) images. Figure 5a–c and d–f presents the TEM results for the water-quenched (WQ) and furnace-cooled (FC) specimens, respectively. The BF image in Figure 5a reveals a microstructure similar to that shown in Figure 5a, consisting of elongated α″ plates with a high density of dislocations. The SAED pattern acquired from the region in Figure 5a is displayed in Figure 5b. Indexing of the SAED pattern confirms the presence of α″ martensite along the [1$\overset{-}{1}$2] zone axis. However, no diffraction spots corresponding to the ω are observed. Figure 5c shows a DF image generated using the α″ spot marked by a red circle in Figure 5b. The bright contrast regions in this image correspond to the α’’ martensitic lath structure.

In contrast, Figure 5d displays the microstructure of the FC specimens, which is characterized by disordered structures comprising bands of varying sizes and orientations. Figure 5e presents the SAED pattern obtained from the region in Figure 5d, corresponding to the α phase along the [000$\overset{-}{1}$] zone axis. The DF image in Figure 5f, generated using the circled spot in the SAED pattern, reveals the lenticular morphology of the α phase.

**Figure 6:**
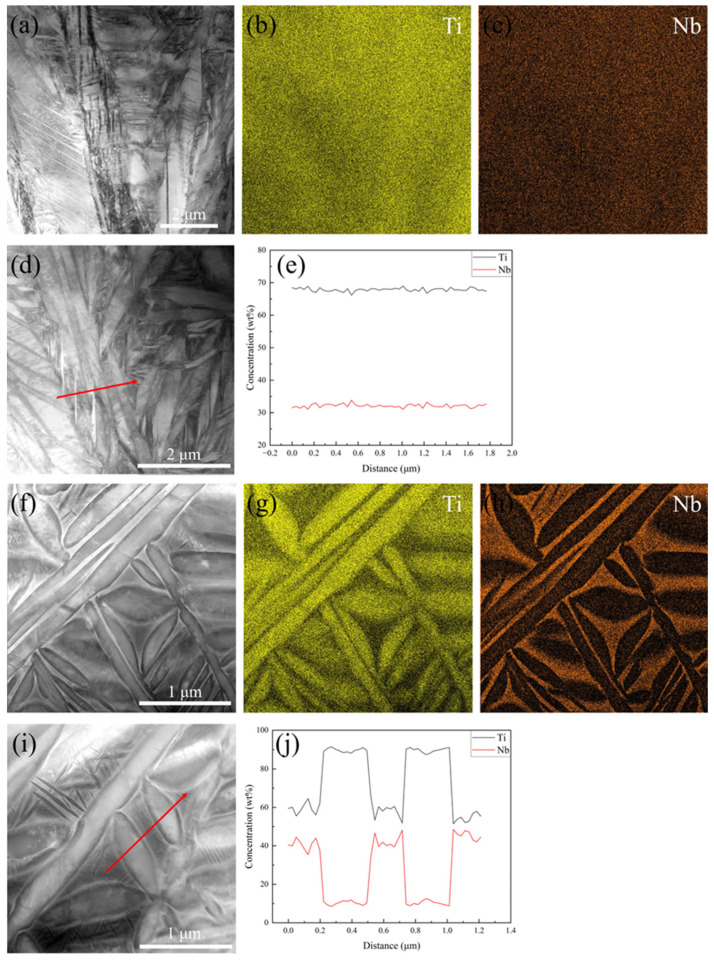
STEM-HAADF images of the Ti-33Nb alloy: (**a**,**d**) WQ; (**f**,**i**) FC. EDS mapping of Ti and Nb in the (**b**,**c**) WQ and (**g**,**h**) FC. (**e**,**j**) Distribution of elements along the red arrows marked in (**d**,**i**).

Figure 6 presents the high-angle annular dark-field (HAADF) images and energy-dispersive X-ray spectroscopy (EDS) mapping of Nb and Ti elements for the water-quenched (WQ) and furnace-cooled (FC) specimens. Representative EDS line scans were performed at specific locations, as indicated in the HAADF images in Figure 6d,i, with the corresponding elemental distribution profiles provided in Figure 6e,j. As shown in Figure 6b,c, Ti and Nb elements are uniformly distributed throughout the WQ specimens, with no evident elemental segregation. In contrast, Figure 6g,h reveals Ti-depleted and Nb-enriched regions, which correspond to the β phase. These observations are consistent with EDS line scan results.

Additionally, it is important to note that the Z contrast of HAADF images is directly proportional to the atomic number (Z) of the elements [31]. This contrast mechanism allows for a clear visualization of the segregation phenomenon. In the FC specimens, the β phase regions exhibit bright contrast due to the enrichment of Nb and depletion of Ti. The EDS mapping and line scan results demonstrate that elemental diffusion is negligible in the WQ specimens, whereas during furnace cooling, Nb diffuses from the α phase to the β phase and Ti diffuses from the β to the α phase.

### 3.3. Nanoindentation of Ti-33Nb Alloys

**Figure 7:**
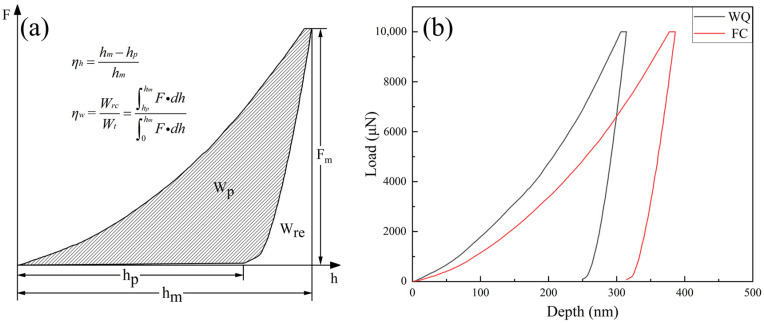
(**a**) Schematic representations of various parameters used for the analysis by nanoindentation load (F)–displacement (h) curve; (**b**) the representative F-h curves of the Ti-33Nb alloys.

The compressive behaviors of the Ti-33Nb specimens under two different heat treatment conditions (furnace cooling and water quenching) were investigated using nanoindentation. To facilitate the analysis of nanoindentation data, Figure 7a provides a schematic representation of the load versus indenter displacement (F-h) curve. During the test, the alloy specimen underwent three main stages. In the loading stage, the specimen first experienced elastic deformation due to the indenter’s action, which gradually transitioned to plastic deformation as the load increased. The indenter penetrated steadily, resulting in a smooth curve. Then, in the load-holding stage, which lasted for 5 s, the purpose was to eliminate the effects of stress–strain hysteresis and creep from the loading stage on the test results. Finally, during the unloading stage, elastic deformation recovered with the reduction in the load, while the plastic deformation remained, forming an indentation on the specimen’s surface. The superelastic response is evaluated based on the F-h curve by calculating the depth recovery ratio (η_h_) and work recovery ratio (η_w_), with the corresponding formulas illustrated in Figure 7a. Here, W_rc_ represents the ability of a superelastic material to absorb deformation energy during indentation without permanent damage, while W_p_ denotes the energy dissipated due to plastic deformation and the reversible movement of phase boundaries during the nanoindentation process [25]. The total energy (W_t_), which is the sum of W_p_ and W_rc_, corresponds to the area enclosed by the loading curve and the maximum penetration depth [32].

As shown in Figure 7b, the Ti-33Nb specimens in both states exhibit non-linear behavior during loading–unloading cycles. Notably, the F-h curve of the furnace-cooled (FC) specimens shifts progressively to the right compared to that of the water-quenched (WQ) specimens, indicating that the nanoindentation depth increases at the same load for specimens subjected to slower cooling rates. This behavior is attributed to the presence of the equilibrium α phase in the FC specimens rather than the martensite α’’ phase, which diminishes the shape memory effect. These results confirm that the FC specimens primarily consist of the equilibrium α phase. In addition, during the rapid cooling process of the water-quenched (WQ) specimens, quenching stresses and a large number of dislocations and other defects are generated [33]. These factors can inhibit the movement of dislocations within the material, thereby enhancing the material’s resistance to deformation. As a result, in nanoindentation tests, a larger load is required to achieve the same displacement. Consequently, the F-h curve of the water-quenched (WQ) alloy exhibits a smaller shift compared to that of the furnace-cooled (FC) specimens.

**Figure 8:**
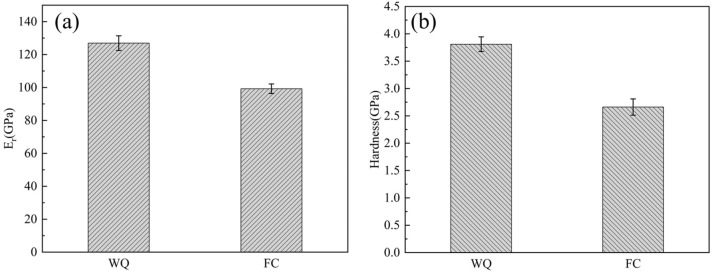
(**a**) The reduced elastic modulus (E_r_) and (**b**) the hardness (H) of the Ti-33Nb alloys.

The reduced elastic modulus (E_r_) and hardness (H) values are summarized in Table 1, while the trends in hardness and elastic modulus are illustrated in Figure 8. The E_r_ value of the furnace-cooled (FC) specimens is 99.24 GPa, which it is lower than that of the water-quenched (WQ) specimens (126.93 GPa). The indentation hardness of both specimens followed a trend similar to that of the E_r_. In multiphase alloys, the elastic modulus is influenced by the moduli of the individual phases and their respective volume fractions. For titanium alloys, the relationship between the phases and their moduli and hardness can be described as E_α_ > E_α″_ > E_β_, H_α″_ > H_β_ > H_α_ [34]. Additionally, the presence of a mixture of β and α″ phases can lead to a reduction in modulus [35]. Therefore, the lower hardness observed in the FC specimens can be attributed to the significant volume fraction of the α phase.

Regarding the elastic modulus, the WQ specimens, which consist of β and α″ phases, would theoretically exhibit a lower elastic modulus compared to the FC specimens composed of β and α phases. However, the relatively higher elastic modulus of the WQ specimens may be explained by the presence of the ω phase.

**Figure 9:**
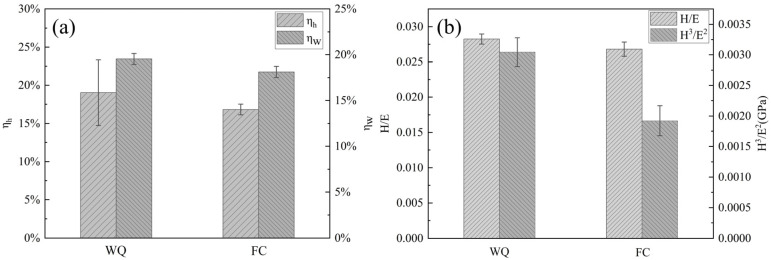
(**a**) The depth recovery ratio (η_h_) and the work recovery ratio (η_w_) of the Ti-33Nb alloys; (**b**) the H/E_r_ ratio and the $H^{3}/E_{r}^{2}$ ratio of the Ti-33Nb alloys.

To evaluate the superelastic behavior of the Ti-33Nb alloys, the depth recovery ratio (η_h_) and work recovery ratio (η_w_) are presented in Figure 9a, with their corresponding values listed in Table 1. The η_h_ and η_w_ values for the water-quenched (WQ) specimens are 19.02% and 19.54%, respectively, which is significantly higher than those of the furnace-cooled (FC) specimens. This indicates that the WQ specimens exhibit superior superelasticity. Superelasticity is primarily attributed to the reversible stress-induced martensitic transformation [36]. During the initial contact between the indenter and the specimens, elastic deformation occurs. As the applied stress increases beyond the critical stress required for martensitic transformation, the β phase within the specimens undergoes stress-induced martensitic transformation into α″ martensite. With further stress application, the pre-existing α’’ martensite in the specimens reorients, followed by the onset of plastic deformation. Upon unloading, the indentation caused by plastic deformation remains, while a portion of the α’’ martensite reverts to the β phase through reverse martensitic transformation, leading to partial recovery of the deformation. Consequently, the WQ specimens, which contain a significant volume fraction of the β phase, demonstrate enhanced superelastic properties.

In addition to the parameters discussed above, nanoindentation can also be utilized to evaluate the wear resistance and anti-wear properties of materials [37]. Wear resistance is closely related to a material’s ability to withstand elastic strain before failure, which is quantified by the H/E_r_ ratio [38]. A higher H/E_r_ value corresponds to greater wear resistance. Figure 8b illustrates the H/E_r_ ratios of the Ti-33Nb alloys. The H/E_r_ value for the water-quenched (WQ) specimens is 0.0282, while that for the furnace-cooled (FC) specimens is 0.0262, indicating that the WQ specimens exhibit superior wear resistance. Furthermore, the H/E_r_ values of the Ti-33Nb alloys under both heat treatment conditions are higher than that of commercially pure titanium (cp-Ti, 0.0238) [39], demonstrating that the Ti-33Nb alloys possess better wear resistance and potential longer service life as biomedical materials.

The resistance to plastic deformation under loading can be effectively evaluated using the $H^{3}/E_{r}^{2}$ ratio (sometimes referred to as yield pressure) [40]. A higher $H^{3}/E_{r}^{2}$ value indicates a greater ability to resist plastic deformation. Figure 9b shows the $H^{3}/E_{r}^{2}$ ratios of the Ti-33Nb alloys. The $H^{3}/E_{r}^{2}$ values for the water-quenched (WQ) and furnace-cooled (FC) specimens are 0.0030 GPa and 0.0019 GPa, respectively. This demonstrates that the WQ specimens with a higher $H^{3}/E_{r}^{2}$ value exhibit superior resistance to plastic deformation. Additionally, the $H^{3}/E_{r}^{2}$ values of both Ti-33Nb alloys are higher than that of commercially pure titanium (cp-Ti, 0.0014 GPa) [39], suggesting that the Ti-33Nb alloys possess enhanced resistance to plastic deformation compared to cp-Ti.

## 4. Conclusions

Ti-33Nb alloy specimens were solution-treated and subsequently cooled by furnace cooling (FC) and water quenching (WQ). Investigations into the influence of stable and metastable phase transformations under different heat treatment conditions on the microstructure and mechanical properties of the alloy yielded the following conclusions:

(1) The microstructure analysis indicates that the WQ specimens, subjected to a higher cooling rate, consist predominantly of β and α″ phases, whereas the FC specimens, cooled at a slower rate, are characterized by the presence of β and α phases.
(2) The nanoindentation test results reveal that the hardness of the FC specimens is significantly lower than that of the WQ specimens, primarily due to the presence of a substantial volume fraction of α phases in the FC specimens. Furthermore, the higher elastic modulus observed in the WQ specimens compared to the FC specimens can be attributed to the formation of the ω phase in the WQ specimens.
(3) The indentation depth recovery ratio (η_h_) and work recovery ratio (η_w_) were derived from the analysis of the P-h curves. The findings demonstrate that the superelastic behavior of the water-quenched (WQ) specimens is significantly enhanced compared to that of the furnace-cooled (FC) specimens. Specifically, the H/E_r_ and $H^{3}/E_{r}^{2}$ ratios of the WQ specimens were determined to be 0.0282 and 0.0030 GPa, respectively. These results suggest that the WQ specimens exhibit superior wear resistance and a higher resistance to plastic deformation relative to both the FC specimens and commercially pure titanium (cp-Ti).

## Figures and Tables

**Table 1:** Measured mechanical properties of Ti-33Nb alloys from the nanoindentation, including hardness (H), reduced elastic modulus (E_r_), H/E_r_, $H^{3}/E_{r}^{2}$, depth recovery ratio (η_h_), and work recovery ratio (η_w_).

Samples	E_r_ (GPa)	H (GPa)	η_h_ (%)	η_w_ (%)	H/E_r_	$H^{3}/E_{r}^{2}$ (GPa)
WQ	126.93	3.81	19.02	19.54	0.0282	0.0030
FC	99.24	2.66	16.82	18.12	0.0268	0.0019

## Data Availability

The original contributions presented in this study are included in the article. Further inquiries can be directed to the corresponding author.
